# Multiple plaque incisions with or without grafting for Peyronie's disease

**DOI:** 10.1002/bco2.130

**Published:** 2022-01-12

**Authors:** Paul K. Hegarty, Daniel O Sullivan, Penelope A. Hegarty, Helen Zafirakis

**Affiliations:** ^1^ Total Urology Affidea Diagnostics Cork Ireland; ^2^ Department of Medicine University College Cork Cork Ireland

**Keywords:** IIEF, incision, Peyronie's disease, plaque, PROM

## Abstract

**Objectives:**

To assess novel surgical techniques in management of Peyronie's disease.

**Subjects:**

Forty‐three men underwent corrective surgery using either partial plaque incision and nongraft (PPING) or multiple plaque incisions and graft (MPIG). The technique used was determined intra‐operatively. Patients were assessed at baseline and follow‐up based on Peyronie's disease questionnaire patient‐reported outcome measure (PDQ‐PROM) and erectile function.

**Results:**

The two groups were well matched in age and erectile function. At baseline MPIG group had greater deformity and poorer patient‐reported outcome. Penile curvature improved from 67.9° to 10.5° in the PPING group and 77.9° to 7.1° with MPIG. PDQ‐PROM improved from 29 to 13 in those who underwent PPING and 38.5 to 17.6 in those undergoing MPIG. Erectile function was preserved in both groups.

**Conclusions:**

These novel surgeries are effective in restoring penile shape and length while preserving erectile function. This is reflected in improved patient‐reported outcomes. These findings should be verified by multi‐institutional study.

## 1 INTRODUCTION

Peyronie's disease (PD) is a common but poorly understood condition. Incidence increases with age and affects 3%–20% of men depending how incidence is studied.^1^, ^2^ Fibrosis of the tunica albuginea may cause deformity and shortening of the erection to the extent of limiting or precluding penetrative intercourse. The management of PD ranges from oral agents^3^, ^4^ in the acute phase to traction therapy,^5^ plaque injection,^6^, ^7^ and various surgeries.^8^, ^9^, ^10^, ^11^ The two main strategies of corrective surgery are either to shorten the convex side diametrically opposite the site of deformity or to incise the plaque and graft to eliminate the concavity. These have inherent limitations. Shortening the convexity is thought to reduce erect length by 1 cm for every 30° corrected. This can be achieved by excising an ellipse such as in the Nesbit,^8^ use of the Heineke–Mikulicz principle as in the Yachia,^9^ or the use of plicating sutures.^10^ Incision and grafting is described by Lue^11^ whereby the plaque is typically incised transversely with extension longitudinally or obliquely at each end of the transverse incision to create a rectangular defect in the tunica albuginea where a graft such as vein or xenograft is applied. This can achieve straightening of the erection without loss of length; however, erectile dysfunction is reported in up to 45% of men undergoing this surgery.^11^

As the tunica albuginea has two layers, inner circular and outer longitudinal, the concept of making multiple partial thickness incisions transversely across the plaque was developed. For patients in whom this is insufficient to straighten, full thickness incisions can be made. Collagen fleece (*TachoSil*, Takeda Pharmaceutical Company Limited) has been used to bridge defects when inserting a penile prosthesis.^12^, ^13^, ^14^ We have developed this further to cover multiple defects on a functional penis without need for suturing. The concept of multiple transverse incisions is to limit the damage to subtunical veins and smooth muscle thereby protecting erectile function. We describe a prospective series of men undergoing these surgeries. The aim is to assess the surgery in terms of patient satisfaction and measurement of deformity and length. The two procedures are described together as the decision for the need to graft or not is made intra‐operatively on an intention to treat basis.

### 1.1 Population

Ethical approval for this study was granted by Clinical Research Ethics Committee of the Cork Teaching Hospitals, University College Cork, Ireland. Between February 2015 and February 2021, 43 men consented to undergo surgery. All patients were instructed on use of the vacuum erectile device and selected for inclusion if they were content in its use and committed to use for 3 months after surgery. Exclusion criteria were men who were unable to perform vacuum therapy or those for whom severe erectile dysfunction warranted insertion of penile prosthesis. Calcification/ossification was not a contraindication. All patients filled the Peyronie's disease patient‐reported outcome measure (PD‐PROM)^15^ prior to surgery and at 3 monthly time points. Twenty‐five (mean age 54, range 31–67 years old) underwent partial plaque incision and nongraft (PPING). Eighteen men (mean age 54, range 29–69 years old) underwent multiple plaque incisions and graft (MPIG) procedures. Erect length and curvature were recorded at the start and end of the procedure by the single surgeon. Patients were also instructed on how to model the penis in the flaccid state in the initial days after surgery when vacuum therapy was not feasible. Vacuum therapy was used so as to maintain the benefit of surgery. Traction therapy was not used perioperatively so as to not confound the results of surgery. All data was entered on an *Excel* database. Two‐tailed Student *t*‐tests were used for comparison of pre‐operative and postoperative data. Statistical significance was defined as *p* < 0.05.

### 1.2 Surgical technique

**FIGURE 1:**
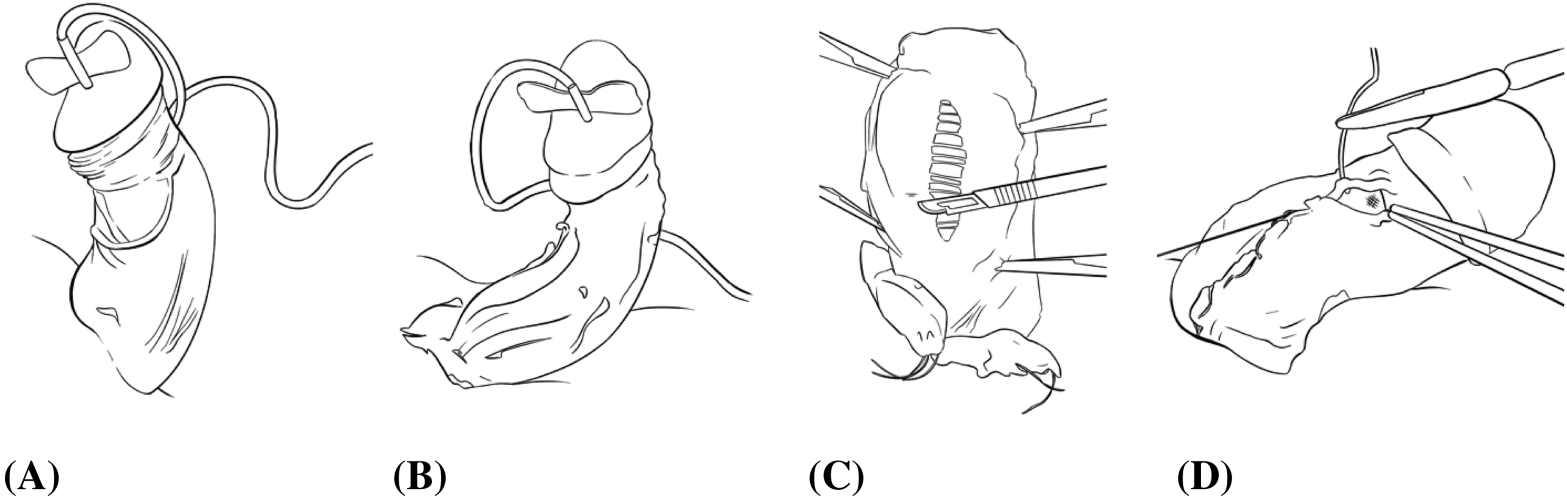
(A) Artificial erection. (B) Degloved penis. (C) Dorsal view, Buck's fascia has been mobilized. Transverse incisions of the plaque are confined to the outer layer of the tunica albuginea. (D) Closure of Buck's fascia

All cases were under general anesthetic in the supine position. An artificial erection was established using saline via a butterfly needle placed through the glans penis into one corpus cavernosum to confirm the curvature prior to degloving the penis (Figure 1A). Once degloved (Figure 1B **)**, the erect length from pubis to meatus was recorded along with the curvature using a goniometer. Buck's fascia was mobilized via the dorsal midline over the deep penile vein in most case where the target plaque was predominantly dorsal or paraurethrally if mostly ventrally located. Exposure was to include normal tunica for at least 1 cm beyond plaque. The plaque was then marked with a surgical marking pen.

### 1.3 Partial plaque incision Nongraft

Multiple transverse incisions 2–3 mm apart were made with a size 15 scalpel transversely through the plaque and extended into normal tunica albuginea for up to 1 cm (Figure 1C). These were made shallow enough to preserve the inner circular fibers. If there was a small breach into the corpus cavernosum, this was closed with a horizontal mattress suture of 2/0 polyglactin. Larger breaches required grafting and reflected a thicker plaque involving the inner tunical layer. As the inner layer is preserved, it is easy to frequently check the gradual correction of the deformity. Provided the curvature was reduced to 10° or less, there was no need to progress to MPIG. Buck's fascia was closed with 3/0 polyglactin running suture. If there was any concern about hemostasis of that layer, a liquid haemostatic agent (*Surgiflo*, from Ethicon) was applied deep to Buck's fascia prior to passing the final sutures (Figure 1D **)**. The penile layers were restored with closure of Dartos and skin separately. If foreskin was present, it was preserved in all cases. Penis was dressed with paraffin imbedded gauze to the incision, soft gauze to the shaft skin, and compressive bandage applied. The patient was discharged the same day and advised to keep the compressive bandage for 48 h. Then he was to start penile modeling of the flaccid penis once daily and vacuum therapy twice daily for 5 min for 3 months once pain and bleeding had subsided. Men were instructed to encourage natural erections as soon it was comfortable but to avoid intercourse/masturbation for 6 weeks.

### 1.4 Multiple plaque incisions and graft

**FIGURE 2:**
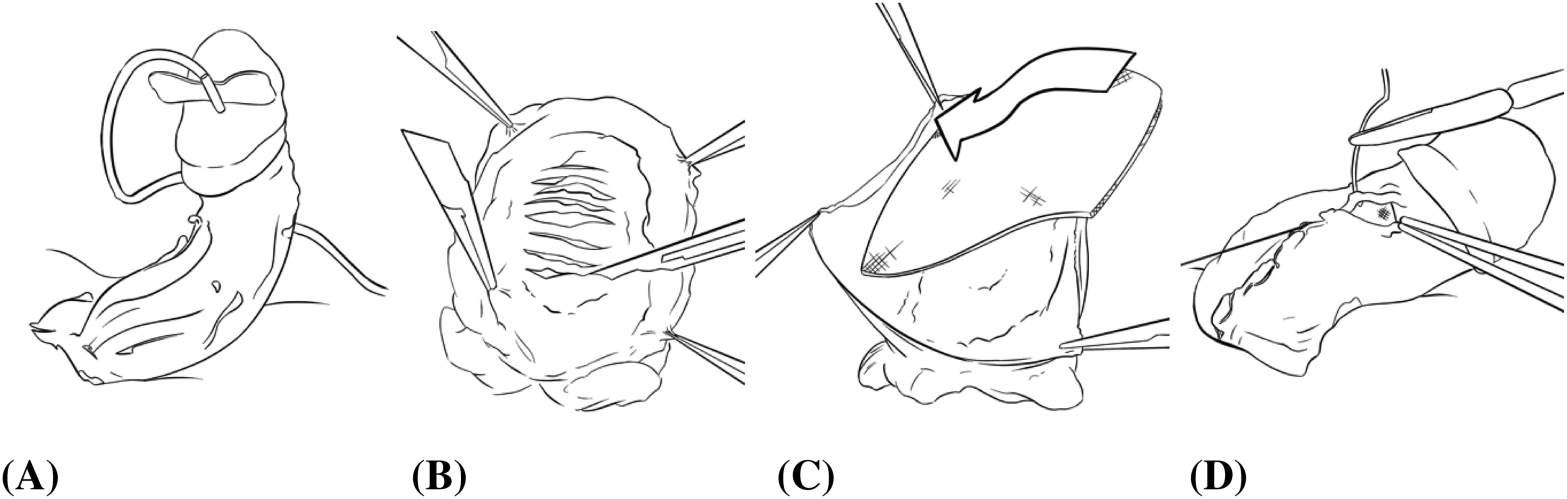
(A) Degloved penis. (B) Full thickness transverse incisions are made through tunica albuginea, while preserving underlying veins and erectile tissue. (C) Collagen fleece covers full thickness tunical defects, placed deep to Buck's fascia. (D) Closure of Buck's fascia

These were cases where the PPING was insufficient to straighten the penis or where there was complexity such as waist deformity. The decision to proceed to MPIG was made intra‐operatively (Figure 2A **)**. Here the incisions are again 2–3 mm apart made through the plaque with a size 15 scalpel (Figure 2B). If there was a waist deformity, the incisions may have been oblique or longitudinal to restore the shape. The concept was not to excise any of the tunica thereby protecting subtunical veins and erectile tissue and to provide a scaffold over which the fleece is applied. The collagen fleece was then applied with a 1‐cm margin beyond all incised areas (Figure 2C). Manufacturer's instructions were followed by applying it dry without touching and activating with a damp sponge for 2 min. Buck's fascia is closed over this in the same way as the PPING (Figure 2D), followed closure of Dartos fascia and skin. Supporting the applied graft with an overlying sponge allows for checking of the final shape and if necessary further incisions made. Dressing and postoperative care is the same as the PPING.

## 2 RESULTS

The pre‐operative findings are summarized in Table 1. The groups were similar in age. Those undergoing MPIG had a statistically significant greater deformity and poorer PDQ‐PROM compared with those who underwent PPING.

**TABLE 1:** Patient demographics prior to corrective surgery

	PPING	MPIG	Total	*P* value
Age (years)	54.7 (8.4)	54.2 (11.2)	54. (9.6)	0.87
Penile curvature (degrees)	67.9 (20.2)	77.9 (17.7)	72.1 (19.6)	0.09
Penile length (cm)	10.2 (2.7)	10.1 (2.2)	10.1 (2.4)	0.89
PDQ‐PROM	29.0 (11.3)	38.5 (11.8)	32.3 (12.2)	0.04
IIEF‐5	20.0 (4.6)	20.4 (5.1)	20.1 (4.73)	0.83

*Note*: Standard deviation is in brackets.

Abbreviations: MPIG, multiple plaque incisions and graft; PPING, partial plaque incision and nongraft.

**FIGURE 3:**
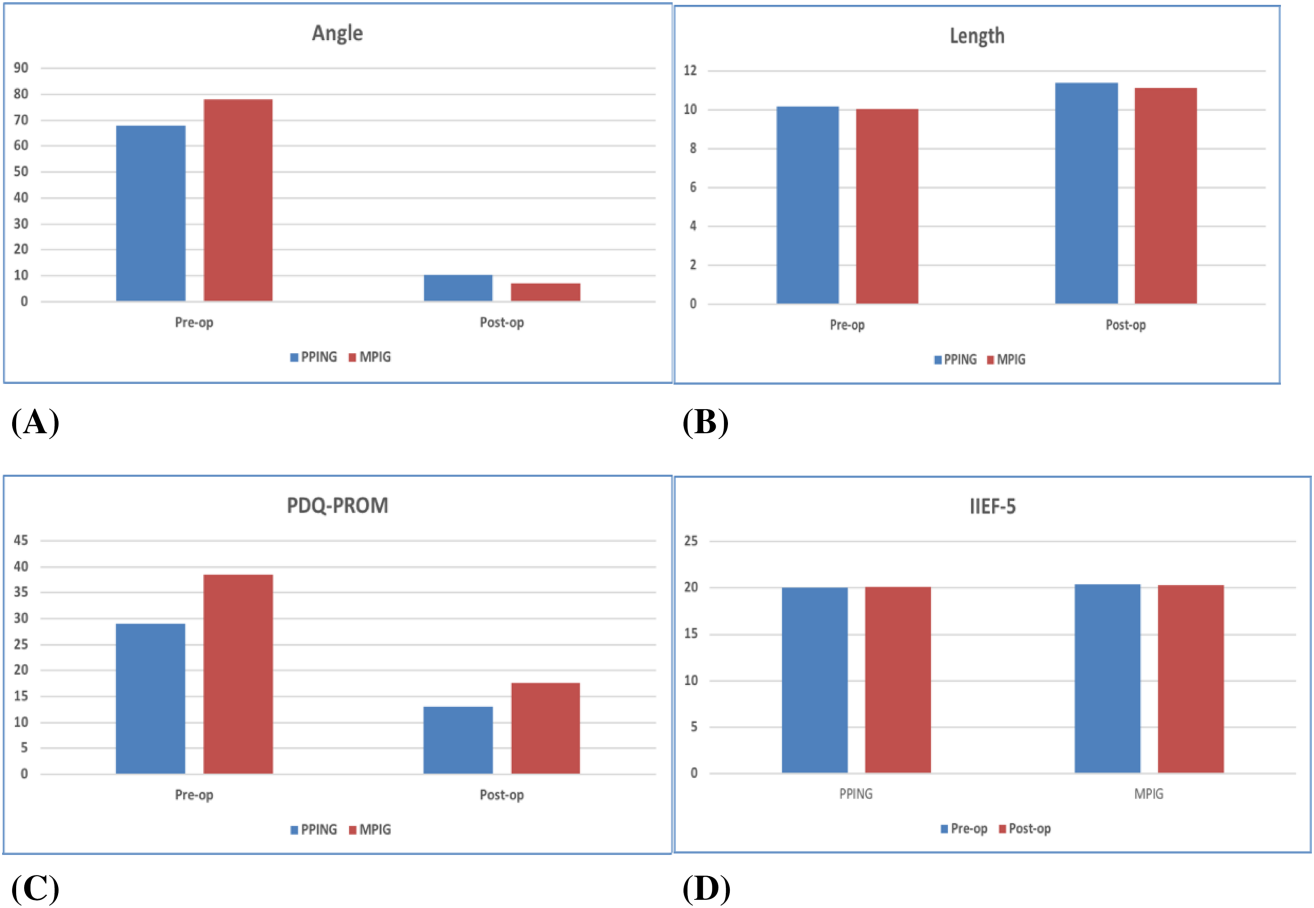
(A) Penile angle (degrees) pre‐operatively and postoperatively for both procedures. (B) Penile erect length (cm) pre‐operatively and postoperatively for both procedures. (C) Patient‐reported outcome pre‐operatively and postoperatively for both procedures. (D) Erectile function outcome pre‐operatively and postoperatively for both procedures

The outcome of surgery is described in Table 2 in terms of penile length, curvature, and PD‐PROM for both procedures and the group as a whole. Follow‐up was for a median of 30 months for the overall group, a median of 37 months for the PPING, and 26 months for MPIG (standard deviation of 15, 14, and 14 months, respectively). Mean penile curvature went from 67.9° to 10.5° with the PPING and from 77.9° to 7.1° with the MPIG (Figure 3A). Mean erect length increased from 10.2 to 11.4 cm with the PPING and from 10.1 to 11.1 cm in the MPIG group (Figure 3B). PDQ‐PROM changed from 29.9 to 13.0 with PPING and 38.5 to 17.6 with MPIG (Figure 3C). Following surgery, PDQ‐PROM was stable through the duration of the study. Two patients in the MPIG group initially had straight erections. The condition reactivated, and new deformity occurred away for the original curvature. Both were re‐operated with a MPIG (Clavien–Dindo Grade IIIB). One of these maintained the benefit; the second had recurrence of curvature and remains dissatisfied, though he can achieve penetrative intercourse. There was one Clavien–Dindo Grade I complication which was one patient who described temporary numbness of part of the glans that resolved within 3 months. Erectile function was maintained in both groups (Figure 3D).

**TABLE 2:** Outcomes of surgery

	Pre‐operative	Postoperative	*P* value
Penile curvature PPING (degrees)	67.9	10.5	<0.05
Penile curvature MPIG (degrees)	77.9	7.1	<0.05
Penile curvature Total (degrees)	72.8	9.3	<0.05
Penile length PPING (cm)	10.2	11.4	<0.05
Penile length MPIG (cm)	10.1	11.1	<0.05
Penile length Total (cm)	10.0	11.3	<0.05
PDQ‐PROM PPING	29.0	13.0	<0.05
PDQ‐PROM MPIG	38.5	17.6	<0.05
PDQ‐PROM Total	32.3	12.2	<0.05
IIEF‐5 PPING	20.0	20.1	n/s
IIEF‐5 MPIG	20.4	20.3	n/s
IIEF Total	20.1	20.2	n/s

Abbreviations: MPIG, multiple plaque incisions and graft; PPING, partial plaque incision and nongraft.

## 3 DISCUSSION

The outcomes of reconstructive surgery are predominantly reported as case series. There are a few studies with level one evidence in PD, such as the use of Potaba in the acute phase of the condition^3^ or collagenase injections.^6^, ^7^ It is more appropriate that patient‐reported outcomes are being used increasingly. There are several surgical options that either shorten the penis or expose the man to potential loss of erectile function. The two techniques described here are applicable without apparent loss of length or function. Patient‐reported outcomes are excellent with 42 of the 43 being satisfied with the outcome. This technique is novel in the use of multiple incisions, with or without grafting over a still function penis. Collagen fleece has been used before to restore length and girth while insertion a penile prosthesis with excellent outcomes.^12^, ^13^, ^14^ Success with these techniques has allowed application to the penis with preserved erectile function.

There are several weaknesses of this study. Firstly, this series does not have a comparison group. The two subgroups are not randomized; rather, they are selected intra‐operatively. The series represents a single surgeon's experience and ought to be replicated by other orthophallic surgeons. Secondly, neither the surgeon nor the patients were blinded as to the treatment. Thirdly, patients used vacuum erectile device during rehabilitation rather than traction devices. Vacuum was chosen so as to maintain the benefit of surgery during healing rather than traction which may confound the results by extending the penis further. We used vacuum therapy to prevent relapse in the 3 months after surgery. It remains to be seen whether any vacuum therapy or traction is required in future studies. Fourth, only angles and lengths were measured intra‐operatively as these are difficult to reproduce confidently in the clinic. These measurements are used by the surgeon intra‐operatively, whereas questionnaires are the domain of the patient.

The strengths are that patient‐reported outcomes are used and that follow‐up is up to 5 years. Nonetheless, this work ought to be compared with conventional surgery such as the Nesbit^8^ and Lue^11^ procedures as part of a multi‐institutional randomized study.

## 4 CONCLUSION

These surgeries are effective in restoring penile shape and length without compromise of erectile function. This is reflected in improved patient‐reported outcomes when compared with their scores at baseline. These findings should be verified by multi‐institutional study.

## CONFLICT OF INTEREST

None.
